# Presymptomatic type 1 diabetes and disease severity at onset. Reply to Schneider J, Gemulla G, Kiess W et al [letter]

**DOI:** 10.1007/s00125-023-06017-z

**Published:** 2023-09-19

**Authors:** Sandra Hummel, Nadine Friedl, Christiane Winkler, Anette-G. Ziegler, Peter Achenbach, for the Fr1da Study Group

**Affiliations:** 1 Institute of Diabetes Research, Helmholtz Munich, German Research Center for Environmental Health, Munich, Germany; 2 German Center for Diabetes Research (DZD), Munich, Germany; 3 Forschergruppe Diabetes e.V. at Helmholtz Zentrum München, Munich, Germany; 4 Forschergruppe Diabetes at Klinikum rechts der Isar, School of Medicine, Technical University Munich, Munich, Germany

**Keywords:** Children, Early-stage diagnosis, Islet autoantibodies, Screening, Type 1 diabetes

*To the Editor:* We thank Schneider and colleagues for their comments [1] on our article ‘Children diagnosed with presymptomatic type 1 diabetes through public health screening have milder diabetes at clinical manifestation’ [2] and appreciate the opportunity to respond to this letter. The authors reported data on diabetic ketoacidosis (DKA), clinical symptoms and hospitalisation at clinical manifestation of type 1 diabetes in children from the Childhood Diabetes Registry of Saxony, who did not participate in an islet autoantibody screening programme. They compared these data with the outcomes we reported in children from the Fr1da study, who were diagnosed with presymptomatic type 1 diabetes prior to clinical onset [2]. We agree that consideration of patient-relevant clinical endpoints is important for the evaluation of islet autoantibody screening programmes. Therefore, the demonstration of lower rates of DKA and symptoms and shorter hospital stays in children from the Fr1da study compared with children in the Childhood Diabetes Registry of Saxony underscores the benefits of screening [1].

In addition to our original report, and using the same study population [2], we have now also assessed whether presentation with any symptoms and weight loss at clinical type 1 diabetes manifestation differed between children without pre-diagnosis (Bavarian DiMelli registry [3]) and children with an earlier diagnosis of presymptomatic diabetes (Fr1da study [4]). Consistent with data from the Childhood Diabetes Registry of Saxony [1], 621 of 681 (91.2%) children in the Bavarian DiMelli registry had symptoms for a median duration of 28 days (IQR: 19–39 days). In comparison, 53 of 121 (43.8%) children in the Fr1da study had symptoms for a median duration of 6 days (IQR: 2–14 days; *p*<0.001 vs the DiMelli study). Weight loss prior to clinical onset was reported in 487 of 584 (83.4%) children in the DiMelli registry, with an overall median weight loss of 2.0 kg (IQR: 1.0–3.0 kg), compared with 6 of 93 (6.5%) children in the Fr1da study, with an overall median weight loss of 0.0 kg (IQR: 0.0–0.0 kg; *p*<0.001 vs the DiMelli study). The *p* values reported above for symptom duration and weight loss were obtained from regression analysis on impact of Fr1da vs DiMelli, adjusted for sex, having a first-degree relative with type 1 diabetes, age and calendar year at clinical type 1 diabetes diagnosis, IBM SPSS Statistics, version 28 (IBM, Armonk, NY, USA).

In conclusion, together with the findings of Schneider et al [1], our additional findings support the usefulness of population-based screening for islet autoantibodies and follow-up of children with presymptomatic type 1 diabetes to improve patient-relevant clinical outcomes.

## Data Availability

The de-identified individual participant data that underlie the results reported in this letter can be shared between 9 and 36 months after publication of the letter. Requests will be honoured from researchers who provide a methodologically sound proposal and who complete a Data Use Agreement with Helmholtz Munich. Requests should be directed by email to the corresponding authors.

## Abbreviation

DKA: Diabetic ketoacidosis
